# Supplementary material for: FERN – a Java framework for stochastic simulation and evaluation of reaction networks
Source: BMC Bioinformatics. 2008 Aug 29;9:356. doi: 10.1186/1471-2105-9-356 (PMC2553347; doi:10.1186/1471-2105-9-356)
Supplement: Additional file 1 — FERN distribution, Version 1.3. This archive contains the FERN source code and binaries as well as documentation and example models in FernML and SBML. [file 1471-2105-9-356-S1.zip › fern/doc/javadoc/overview-tree.html]

Class Hierarchy


---


|  |  |  |
| --- | --- | --- |
| **Overview** \| Package \| Class \| Use \| **Tree** \| **Deprecated** \| **Index** \| **Help** | |  |
| PREV   NEXT | **FRAMES**    **NO FRAMES**     **All Classes** |  |


---


## Hierarchy For All Packages

**Package Hierarchies:**: fern, fern.analysis, fern.benchmark, fern.cellDesigner, fern.cellDesigner.ui, fern.cytoscape, fern.cytoscape.ui, fern.example, fern.network, fern.network.creation, fern.network.fernml, fern.network.modification, fern.network.sbml, fern.simulation, fern.simulation.algorithm, fern.simulation.controller, fern.simulation.observer, fern.tools, fern.tools.functions, fern.tools.gnuplot

---

## Class Hierarchy

- java.lang.**Object**
  - javax.swing.**AbstractAction** (implements javax.swing.Action, java.lang.Cloneable, java.io.Serializable)
    - cytoscape.util.CytoscapeAction
      - fern.cytoscape.**CytoscapeVisualizer.ShowMainFrameAction**- fern.cytoscape.**CytoscapeVisualizer.SimulationAction**
    - fern.network.**AbstractKineticConstantPropensityCalculator** (implements fern.network.PropensityCalculator)
      - fern.network.**ArrayKineticConstantPropensityCalculator**
      - fern.network.**AbstractNetworkImpl** (implements fern.network.Network)
        - fern.network.creation.**AutocatalyticNetwork** (implements fern.network.creation.CatalystIterator)- fern.cellDesigner.**CellDesignerNetworkWrapper**- fern.cytoscape.**CytoscapeNetworkWrapper**- fern.network.fernml.**FernMLNetwork**- fern.network.sbml.**SBMLNetwork**
        - fern.network.**AmountManager**- fern.analysis.**AnalysisBase**
            - fern.analysis.**AutocatalyticNetworkDetection**- fern.analysis.**ShortestPath**
            - fern.simulation.controller.**AndController** (implements fern.simulation.controller.SimulationController)- fern.network.**AnnotationManagerImpl** (implements fern.network.AnnotationManager)- fern.example.**AutocatalyticNetworkExample**- fern.tools.gnuplot.**Axes** (implements java.lang.Iterable<T>)
                    - fern.tools.gnuplot.**ArrayMatrixAxes**
                      - fern.tools.gnuplot.**TransposedArrayMatrixAxes**
                      - fern.tools.gnuplot.**CollectionAxes**
                    - fern.benchmark.**Benchmark**
                      - fern.benchmark.**RandomNumber**- fern.benchmark.**SimulatorPerformance**
                          - fern.benchmark.**SimulatorCorrectness**- fern.benchmark.**SimulatorTime**
                              - fern.benchmark.**SimulatorFireTypes**- fern.benchmark.**SimulatorRandomNumbers**
                      - CellDesignerPlugin
                        - fern.cellDesigner.**FernCellDesignerPlugin**
                        - fern.cytoscape.**ColorCalculator** (implements java.lang.Cloneable)- fern.tools.**ColorSpectrum**- java.awt.**Component** (implements java.awt.image.ImageObserver, java.awt.MenuContainer, java.io.Serializable)
                              - java.awt.**Container**
                                - javax.swing.**JComponent** (implements java.io.Serializable)
                                  - javax.swing.**JPanel** (implements javax.accessibility.Accessible)
                                    - fern.cellDesigner.ui.**ExtendedPane**- fern.cytoscape.ui.**ExtendedPane**- fern.cellDesigner.ui.**OverviewPane**- fern.cytoscape.ui.**OverviewPane**
                                  - java.awt.**Window** (implements javax.accessibility.Accessible)
                                    - java.awt.**Dialog**
                                      - javax.swing.**JDialog** (implements javax.accessibility.Accessible, javax.swing.RootPaneContainer, javax.swing.WindowConstants)
                                        - fern.cytoscape.ui.**ColorPicker**
                                      - java.awt.**Frame** (implements java.awt.MenuContainer)
                                        - javax.swing.**JFrame** (implements javax.accessibility.Accessible, javax.swing.RootPaneContainer, javax.swing.WindowConstants)
                                          - fern.tools.gnuplot.**GnuPlot**- fern.cellDesigner.ui.**MainFrame**- fern.cytoscape.ui.**MainFrame**
                              - fern.tools.**ConfigReader**- fern.cytoscape.**CytoscapeAnnotationManager** (implements fern.network.AnnotationManager)- cytoscape.plugin.CytoscapePlugin (implements java.beans.PropertyChangeListener)
                                    - fern.cytoscape.**CytoscapeVisualizer**
                                    - fern.example.**DecayingDimerizingHistogramDistances**- fern.example.**DecayingDimerizingInteractive**- fern.example.**DecayingDimerizingPlots**- fern.simulation.controller.**DefaultController** (implements fern.simulation.controller.SimulationController)- fern.simulation.algorithm.**DependencyGraph**- fern.example.**Dsmts**- fern.example.**ExamplePath**- fern.network.fernml.**FernMLAnnotationManager** (implements fern.network.AnnotationManager)- fern.example.**HistogramDistanceTestSet**- fern.simulation.algorithm.**IndexedPriorityQueue**- fern.example.**IrreversibleIsomerization**- fern.example.**LacYComplete**- fern.example.**LacYHistogramDistances**- fern.example.**LacZ**- fern.example.**MapkBenchmark**- fern.network.sbml.**MathTree**- fern.network.sbml.**MathTree.Node**
                                                                      - fern.network.sbml.**MathTree.ConstLeaf**- fern.network.sbml.**MathTree.GlobalLeaf**- fern.network.sbml.**MathTree.InnerNode**- fern.network.sbml.**MathTree.VarLeaf**
                                                                      - fern.example.**MichaelisMentenKinetic**- fern.network.modification.**ModifierNetwork** (implements fern.network.Network)
                                                                          - fern.network.modification.**CatalysedNetwork**- fern.network.modification.**ExtractSubNetwork**- fern.network.modification.**ReversibleNetwork**
                                                                          - fern.cytoscape.**NetworkChecker**- fern.cytoscape.**NetworkChecker.EdgeClassifierByDirection** (implements fern.cytoscape.NetworkChecker.EdgeClassifier)- fern.cytoscape.**NetworkChecker.EdgeClassifierByIdentifier** (implements fern.cytoscape.NetworkChecker.EdgeClassifier)- fern.cytoscape.**NetworkChecker.NodeClassifierByAnnotation**<T> (implements fern.cytoscape.NetworkChecker.NodeClassifier)- fern.cytoscape.**NetworkChecker.NodeParameter**- fern.network.**NetworkLoader**- fern.tools.**NetworkTools**- cytoscape.visual.NodeAppearanceCalculator
                                                                                          - fern.cytoscape.**CytoscapeColorChangeObserver.ColorChangingNodeAppeareanceCalculator**
                                                                                          - fern.analysis.**NodeCheckerByAnnotation** (implements fern.analysis.NodeChecker)- fern.tools.**NumberTools**- fern.simulation.observer.**Observer**
                                                                                                - fern.simulation.observer.**AmountAtMomentObserver** (implements fern.simulation.observer.GnuPlotObserver, fern.simulation.controller.SimulationController)- fern.example.**CellGrowthObserver**- fern.cytoscape.**CytoscapeColorChangeObserver**- fern.simulation.observer.**FireTypeObserver** (implements fern.simulation.observer.GnuPlotObserver)- fern.simulation.observer.**InstantOutputObserver**- fern.simulation.observer.**IntervalObserver** (implements fern.simulation.observer.GnuPlotObserver)
                                                                                                            - fern.simulation.observer.**AmountIntervalObserver**- fern.simulation.observer.**MultiAmountIntervalObserver**- fern.simulation.observer.**ReactionIntervalObserver**
                                                                                                            - fern.simulation.observer.**LeapObserver** (implements fern.simulation.observer.GnuPlotObserver)- fern.simulation.observer.**RandomNumberGeneratorCallObserver** (implements fern.simulation.observer.GnuPlotObserver)- fern.simulation.observer.**TriggerObserver**
                                                                                                                  - fern.network.sbml.**SBMLEventHandlerObserver**
                                                                                                - fern.simulation.controller.**OrController** (implements fern.simulation.controller.SimulationController)- cern.colt.**PersistentObject** (implements java.lang.Cloneable, java.io.Serializable)
                                                                                                    - cern.colt.list.**AbstractCollection**
                                                                                                      - cern.colt.list.**AbstractList**
                                                                                                        - cern.colt.list.**AbstractIntList** (implements cern.colt.buffer.IntBufferConsumer)
                                                                                                          - cern.colt.list.**IntArrayList**
                                                                                                            - fern.analysis.**IntQueue** (implements fern.analysis.IntSearchStructure)- fern.analysis.**IntStack** (implements fern.analysis.IntSearchStructure)
                                                                                                    - fern.tools.functions.**Probability.Constant** (implements fern.tools.functions.Probability)- fern.tools.functions.**Probability.ReactionProbability** (implements fern.tools.functions.Probability)- fern.example.**SBMLMathTreeTest**- fern.network.sbml.**SBMLPropensityCalculator** (implements fern.network.ComplexDependenciesPropensityCalculator)
                                                                                                            - fern.cellDesigner.**CellDesignerPropensityCalculator**
                                                                                                            - fern.analysis.**ShortestPath.Path**- fern.simulation.**Simulator**
                                                                                                                - fern.simulation.algorithm.**GibsonBruckSimulator**- fern.simulation.algorithm.**GillespieEnhanced**
                                                                                                                    - fern.simulation.algorithm.**AbstractBaseTauLeaping**
                                                                                                                      - fern.simulation.algorithm.**AbstractTauLeapingPropensityBoundSimulator**
                                                                                                                        - fern.simulation.algorithm.**TauLeapingAbsoluteBoundSimulator**- fern.simulation.algorithm.**TauLeapingRelativeBoundSimulator**
                                                                                                                        - fern.simulation.algorithm.**TauLeapingSpeciesPopulationBoundSimulator**
                                                                                                                    - fern.simulation.algorithm.**GillespieSimple**- fern.simulation.algorithm.**HybridMaximalTimeStep**
                                                                                                                - fern.**Start**- fern.tools.**Stochastics**- java.lang.**Throwable** (implements java.io.Serializable)
                                                                                                                      - java.lang.**Exception**
                                                                                                                        - fern.network.**FeatureNotSupportedException**
                                                                                                                      - cytoscape.visual.VisualStyle (implements java.lang.Cloneable)
                                                                                                                        - fern.cytoscape.**FernVisualStyle**

## Interface Hierarchy

- fern.network.**AnnotationManager**- fern.network.creation.**CatalystIterator**- fern.simulation.observer.**GnuPlotObserver**- fern.analysis.**IntSearchStructure**- fern.network.**Network**- fern.cytoscape.**NetworkChecker.EdgeClassifier**- fern.cytoscape.**NetworkChecker.NodeClassifier**- fern.analysis.**NetworkSearchAction**- fern.analysis.**NodeChecker**- fern.tools.functions.**Probability**- fern.network.**PropensityCalculator**
                      - fern.network.**ComplexDependenciesPropensityCalculator**
                      - fern.simulation.controller.**SimulationController**

## Enum Hierarchy

- java.lang.**Object**
  - java.lang.**Enum**<E> (implements java.lang.Comparable<T>, java.io.Serializable)
    - fern.analysis.**NetworkSearchAction.NeighborType**- fern.cytoscape.**ColorCalculator.Scale**- fern.simulation.**Simulator.FireType**

---


|  |  |  |
| --- | --- | --- |
| **Overview** \| Package \| Class \| Use \| **Tree** \| **Deprecated** \| **Index** \| **Help** | |  |
| PREV   NEXT | **FRAMES**    **NO FRAMES**     **All Classes** |  |


---
